# Mortality Risk from Respiratory Diseases Due to Non-Optimal Temperature among Brazilian Elderlies

**DOI:** 10.3390/ijerph18115550

**Published:** 2021-05-22

**Authors:** Ludmilla da Silva Viana Jacobson, Beatriz Fátima Alves de Oliveira, Rochelle Schneider, Antonio Gasparrini, Sandra de Souza Hacon

**Affiliations:** 1 Department of Statistics, Fluminense Federal University, Niterói 24210-201, RJ, Brazil; 2 Brazilian Research Network on Global Climate Change–Rede Clima, São José dos Campos 12227-010 SP, Brazil; sandrahacon@gmail.com; 3 Nacional School of Public Health, Oswaldo Cruz Foundation, Rio de Janeiro 21041-210, RJ, Brazil; beatrizenf@gmail.com; 4 Department of Public Health, Environments and Society, London School of Hygiene & Tropical Medicine, London WC1E7HT, UK; rochelle.schneider@lshtm.ac.uk (R.S.); antonio.gasparrini@lshtm.ac.uk (A.G.); 5 Centre for Statistical Methodology, London School of Hygiene & Tropical Medicine, London WC1E7HT, UK; 6 Forecast Department, European Centre for Medium-Range Weather Forecast, Reading RG29AX, UK; 7 The Centre on Climate Change and Planetary Health, London School of Hygiene & Tropical Medicine, London WC1E7HT, UK

**Keywords:** heat-related mortality, urban area, respiratory outcomes, elderly, climate reanalysis

## Abstract

Over the past decade, Brazil has experienced and continues to be impacted by extreme climate events. This study aims to evaluate the association between daily average temperature and mortality from respiratory disease among Brazilian elderlies. A daily time-series study between 2000 and 2017 in 27 Brazilian cities was conducted. Data outcomes were daily counts of deaths due to respiratory diseases in the elderly aged 60 or more. The exposure variable was the daily mean temperature from Copernicus ERA5-Land reanalysis. The association was estimated from a two-stage time series analysis method. We also calculated deaths attributable to heat and cold. The pooled exposure–response curve presented a J-shaped format. The exposure to extreme heat increased the risk of mortality by 27% (95% CI: 15–39%), while the exposure to extreme cold increased the risk of mortality by 16% (95% CI: 8–24%). The heterogeneity between cities was explained by city-specific mean temperature and temperature range. The fractions of deaths attributable to cold and heat were 4.7% (95% CI: 2.94–6.17%) and 2.8% (95% CI: 1.45–3.95%), respectively. Our results show a significant impact of non-optimal temperature on the respiratory health of elderlies living in Brazil. It may support proactive action implementation in cities that have critical temperature variations.

## 1. Introduction

The process of climate change is directly related to the atmospheric emission of greenhouse gases (GHGs) caused by human activities. All population will be affected by climate change, but the health risks vary, depending on the place and how the exposed people live. Global warming has accelerated in the last decade and has been linked to the increased frequency of extreme weather-related events and the unusual temporal and spatial distribution of precipitation, humidity, and wind speed. Additionally, climate change has caused an increase in global average temperatures. All of these factors have important implications for human health and have motivated the exponential increase in the number of studies on the effect of temperature on health outcomes in the last 10 years [1].

The Intergovernmental Panel on Climate Change (IPCC) has emphasized the importance of studies on the association between temperature and health outcomes, especially concerning the most vulnerable populations. In the report of the IPCC [2], the priority of the Climate and Health theme was reinforced due to projections of a global average temperature increase between 2 and 4.5 °C by the end of the 21st century.

In recent decades, temperature has been identified as a risk factor for communicable and non-communicable diseases, either by direct exposure or indirect exposure caused by environmental imbalance [3]. Studies have been evaluating the effects of temperature on different health outcomes and population subgroups [4,5]. Additionally, extreme temperatures may increase the risk of hospitalizations and deaths due to respiratory diseases, not only because of exposure to heat but also due to exposure to cold temperatures [6,7,8].

The impact of climate variability on populations’ health is widespread; however, some groups are more vulnerable than others [9]. Studies suggest that the elderly are one of the groups most vulnerable to climate change, because of physiological and social factors [10]. Investigations point to an increased risk of mortality as a result of both high temperatures and low temperatures [11].

In Brazil, in 2010, the elderly (60 years old or more) represented approximately 11% of the Brazilian population and projections indicate that by 2060 this age group will represent around 25% of the population (IBGE—https://www.ibge.gov.br/apps/population/projection//index.html, accessed 12 June 2019). Few studies investigated the effects of temperature exposure on respiratory disease in the Brazilian elderly, and they were in the city of São Paulo [12,13,14]. It is important to have evidence for the different regions of Brazil, as they have different environmental, socioeconomic, health, and political characteristics.

Modeling studies related to Brazilian projections of the Eta-HadGEM2S Regional Model show that an increase in average temperatures of 1.5 °C has already taken place in Brazil since the end of 2010 [15]. Over the past decade, Brazil has experienced and continues to be impacted by extreme climate events such as droughts in the southeast, northeast, and northern regions, particularly in the Amazon [16,17]. Additionally, in the south and southeast regions, global warming has affected the average daily temperature and the daily amplitude and few studies have been conducted in Brazil to evaluate the effects of rising temperatures on human health [18,19].

Therefore, this study aimed to evaluate the association between daily average temperature and mortality caused by respiratory diseases among the elderly in the Brazilian capital cities, considering the aging of the Brazilian population as well as the fact that this group is more vulnerable to climate change.

## 2. Materials and Methods

### 2.1. Study Area and Period

We performed an ecological time-series study from 1 January 2000 to 31 December 2017 in the Brazilian capitals. Brazil is divided into 26 states and the Federal District, and each one has a capital. The capital is usually the most important city of the state according to economic, social, and cultural indicators. All of the capital cities and the Federal District were included in the analysis.

### 2.2. Data

Health outcomes were daily counts of deaths due to respiratory diseases in the elderly aged 60 or more, registered in Brazil’s National Unified Health System (SUS). Data were provided by the Brazilian Health Informatics Department (DATASUS) classified as respiratory diseases (J00-J99), according to the Tenth Revision of the International Classification of Diseases (ICD-10).

The exposure variable was the daily mean of 2 m temperature modeled and produced by the *European Centre for Medium-Range Weather Forecasts* (ECMWF) ERA5-Land climate reanalysis. The ERA5-Land reanalysis is available at a grid resolution of 9 km and hourly temporal frequency and we calculated the daily average using the weighted average of the grids covered for each municipality polygon by apportionment methods. Thus, the daily series of temperature for each municipality was obtained by calculating the average of the pixel values weighted by the proportion of the area of the municipality covered by the pixel. The relative humidity was estimated from the daily mean of the temperature and dew point temperature from the ERA5-Land database using the package *HeatStress* of the Program R [20].

### 2.3. Statistical Analysis

The associations between the mean daily temperature and mortality were estimated from a two-stage time series analysis method. In the first stage, a generalized linear regression model with a quasi-Poisson family, combined with a distributed lag non-linear model (DLNM), were performed for each city. Models were adjusted for seasonal and long-term trends using natural cubic splines of the time variable with city-specific degrees of freedom per year, daily relative humidity using a natural cubic spline function with 3 degrees of freedom, and day of the week as an indicator variable [21]. To evaluate non-linear and lag effects of temperature on mortality we used a DLNM. The exposure–response association was modeled using a natural cubic spline with three internal knots placed at the 10th, 75th, and 90th percentiles of the city-specific temperature distribution, regarding changes in the effect estimates at extreme temperatures; and the lag-response association was modeled with a natural cubic spline and three internal knots placed at equally spaced values in the log scale. Lag period was 21 days according to what has been described in other studies [18,19].

Other possibilities for model fitting were evaluated, varying knots placement, lags, and the number of degrees of freedom. The goodness-of-fit was assessed by visual inspection of the residuals and also based on the minimization of a modified Akaike information criteria [22].

In the second stage, a multivariate meta-regression was used to model heterogeneity between cities using city-specific mean temperature and temperature range (maximum–minimum daily temperature) as the meta-predictors. Multivariate Wald test and residual heterogeneity were evaluated through the Cochran Q test and I^2^ statistic. Subsequently, the best linear unbiased prediction (BLUP) was applied [23].

The results were presented as net effects across the 21 days of lag. We present the relative risks with a 95% confidence interval (CI) in each city related to local mean temperature versus minimum mortality temperature (MMT). MMT is the reference value that corresponds to the optimum temperature where the mortality risk is at its minimum, and was derived from best linear unbiased predictions in each location. The pooled overall cumulative exposure–response curve was presented for all of Brazil.

Moreover, the impacts associated with temperature exposure were estimated in terms of the attributable fraction of death [18]. We calculated the total attributable fraction and the contribution related to days with temperatures below (cold effect) or above (heat effect) the city-specific MMT. To evaluate uncertainties, empirical confidence intervals were calculated for the attributable fractions based on Monte Carlo simulations. Extreme cold and heat attributable mortality was also investigated as described by Gasparrini et al. [18].

All analyses were performed in R software (R Foundation for Statistical Computing, Vienna, Austria) [24], version 3.4.1., using the packages *dlnm* [21] and *mvmeta* [23]. Estimated effects with *p* < 0.05 were considered statistically significant.

## 3. Results

**Figure 1:**
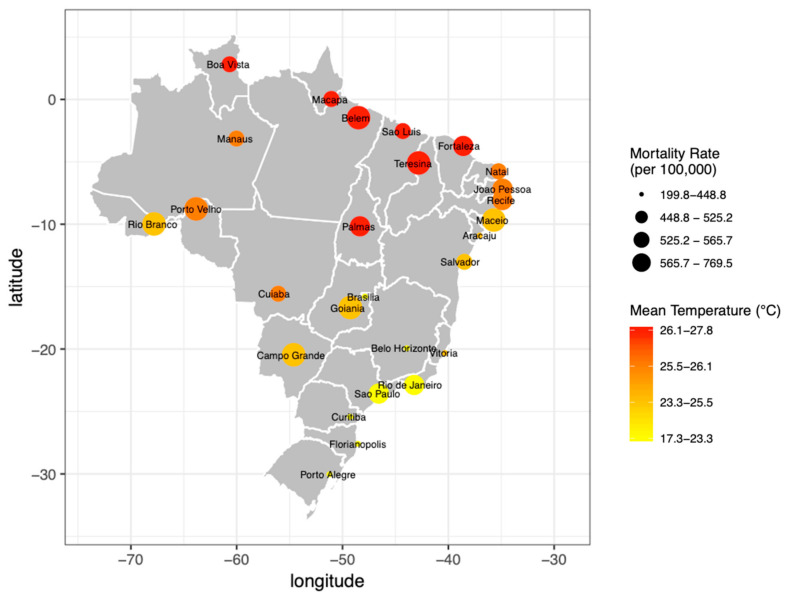
Mean temperature (ERA5-Land) and mortality rate (per 100,000 inhabitants) by capitals. Brazil, from 2000 to 2017.

Figure 1 presents the average mortality rates for respiratory diseases in the elderly and the mean temperature from 2000 to 2017 in the capitals of Brazil. The total number of deaths in Brazil was 422,642. The mortality rates per 100,000 inhabitants were higher in the capitals of Rio Branco (769.5), Belém (743.0), Campo Grande (593.6), Porto Velho (593.6), Maceio (592.9) and Teresina (581.2).

The capitals of the North and Northeast regions had mean daily temperatures around 26 °C, higher than the capitals of the other regions. In the Midwestern region, the average temperatures ranged from 22 to 26 °C, while in the Southeast and South the average temperatures were approximately 22 °C and 19 °C, respectively. As for the temperature range, Porto Alegre was the capital with the largest variation and Fortaleza was the capital with the lowest variability in the period. Table 1 shows descriptive results according to capitals of Brazilian states.

**Figure 2:**
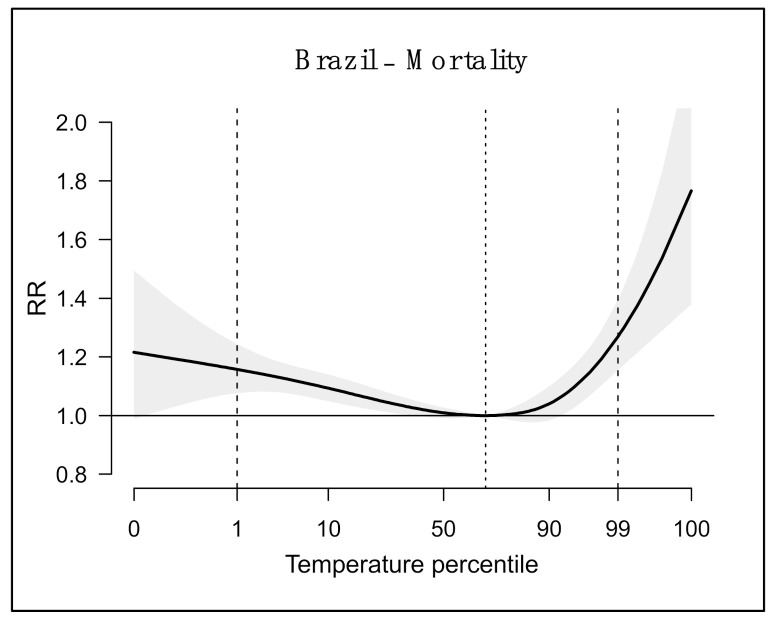
Pooled overall cumulative exposure–response association curve between temperature and mortality. Brazil, capitals, 2000 to 2017. Note: dotted line is the percentile of the minimum risk temperature; dashed lines at 1st and 99th percentiles of the temperature distribution.

Figure 2 shows the pooled exposure–response curve for respiratory deaths in Brazil, which suggest a J-shaped format. The minimum risk temperature in Brazil was 25.4 °C (68th percentile). The exposure to extreme heat (99th percentile vs. minimum mortality percentile—MMP) increased the risk of mortality by 27% (relative risk (RR): 1.27; 95% CI: 1.15–1.39). On the other hand, exposure to extreme cold (first percentile vs. MMP) also increased the risk of mortality (RR = 1.16; 95% CI: 1.08–1.24), but the intensity was smaller.

**Figure 3:**
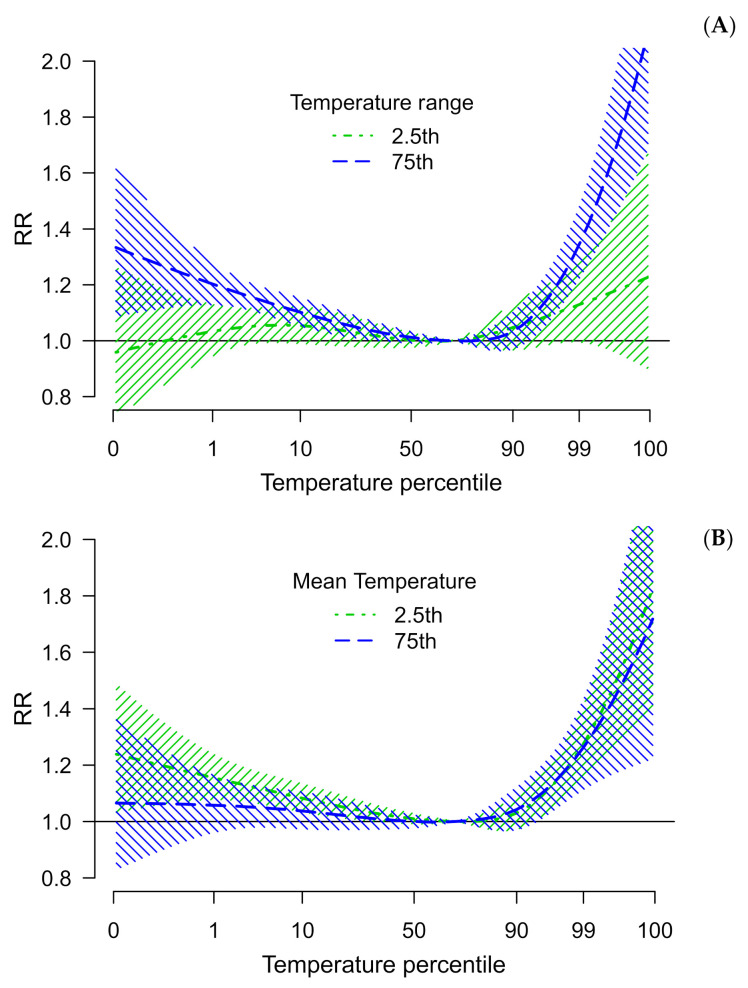
Pooled overall cumulative exposure–response association curve between Table 2. 5th and 75th percentiles of the meta-variables temperature range (**A**) and mean (**B**). Brazil, capitals, 2000 to 2017.

Results from multivariate meta-regression analysis suggest significant residual heterogeneity, even after adjustment for city-specific mean temperature and temperature range (I^2^ = 61.4%). Both predictors significantly modify the temperature mortality association in the full model, but the temperature range accounts for a higher proportion of heterogeneity in single-predictor models (Figure 3A,B).

**Figure 4:**
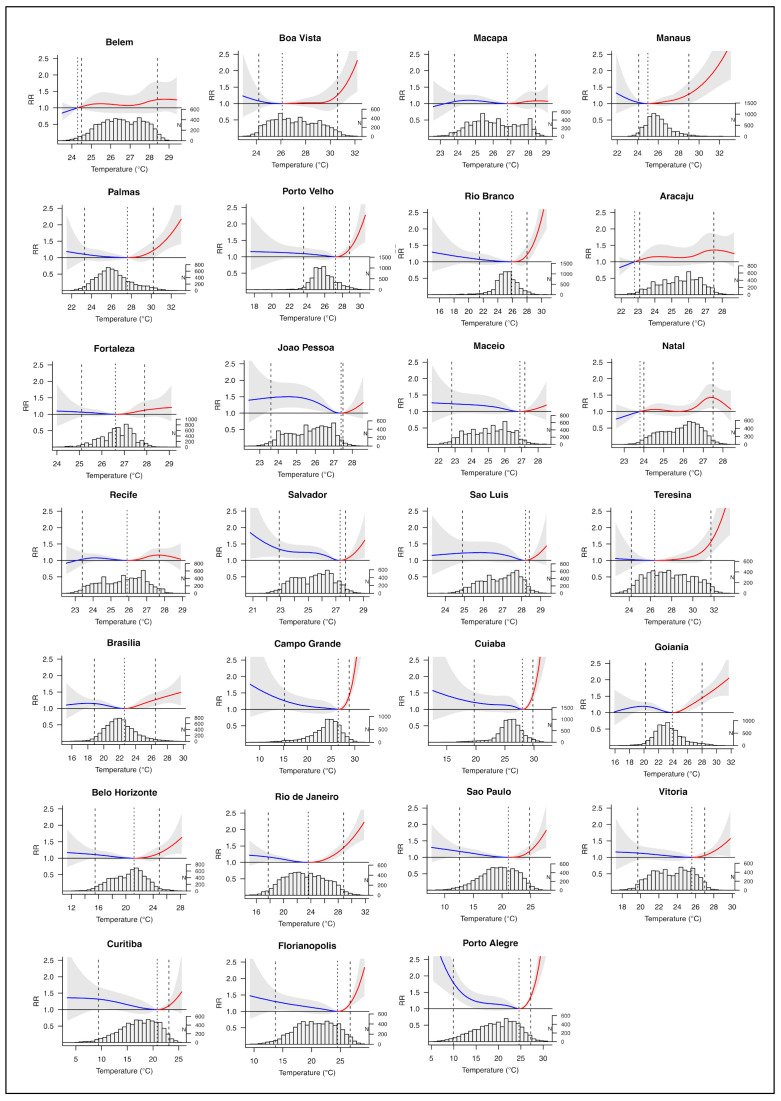
Overall cumulative exposure–response association in Brazilian capitals, 2000 to 2017. Note: exposure–response associations as best linear unbiased prediction (with 95% empirical CI, shaded grey), with related temperature distributions. Solid grey lines are minimum mortality temperatures and dashed grey lines are the 2.5th and 97.5th percentiles. RR = relative risk. *N* = number of deaths.

The exposure–response curves of each capital estimated using BLUP are shown in Figure 4. The overall cumulative effect of 21 days of exposure was significant for all cities of the Southeast region with J-shaped curves, but higher heat effects were observed in Rio de Janeiro. In the South region, the curves were J-shaped for Florianópolis and Curitiba, while for Porto Alegre the estimated curve was U-shaped. All capitals of the Midwest region had significant results and estimated J-shaped curves, with higher heat effects in Campo Grande and Cuiabá. In the North region, most of the capitals presented J-shaped curves and cumulative effects significant only for temperatures above MMT. In the Northeast region, only Teresina presented significant heat effects with a J-shaped curve. On the other hand, Salvador and João Pessoa presented significant cold effects.

Table 2 shows the estimated attributable fraction of mortality and the MMT according to capital cities and Brazil. On average, the MMT was around 25 °C between cities, with the lowest MMT in Curitiba (20.8 °C) and the highest MMT in São Luis (28.2 °C). The total fraction of deaths in Brazil was 7.5% (95% CI: 5.4, 9.2), with the highest attributable risk caused by temperatures below 25.9 °C. This fraction varied widely between capitals, but the greatest values were mainly in the South and Northeast region, highlighting the cities Porto Alegre, João Pessoa, and Salvador. These last capitals also presented the highest cold attributable fractions. As for the fraction attributable to heat, Rio de Janeiro and Goiânia were the capitals with significant and highest fractions, although results gave Aracaju, Belém, Natal and Teresina greater fractions.

The effects of cold and heat were also separated into two components, i.e., moderate and extreme. Extreme cold was related to city-specific temperatures lower than the 2.5th percentile and extreme heat higher was related to temperatures than the 97.5th percentile. In Brazil, the fractions attributable to moderate and extreme cold were 4.3% and 0.4%, respectively. Moreover, the fractions attributable to moderate and extreme heat were 1.8% and 1.1%, respectively. Among cities, Porto Alegre showed the highest percentage fraction attributable to extreme cold and Teresina the highest percentage fraction attributable to extreme heat (Figure S1).

**Figure 5:**
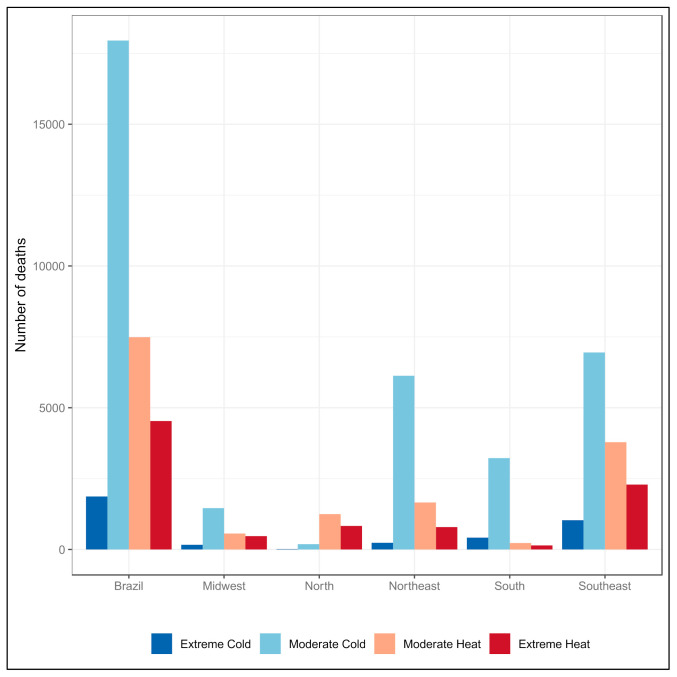
Number of deaths attributable to moderate and extreme temperatures according to region. Brazil, 2000 to 2017.

On the other hand, in terms of the absolute number of deaths attributable to extreme temperatures, the Southeast region had the greatest impact associated with extreme cold (deaths = 1032.9) and also with extreme heat (deaths = 2292.4). São Paulo presented the highest impact of extreme cold (deaths = 596) and Rio de Janeiro the highest impact of extreme heat (deaths = 2276) (Figure 5).

### Sensitivity Analysis

Table S1 and Figure S2 in the Supplementary Materials show the total attributable fraction calculated for each capital and the entire Brazil, according to different model adjustments. In these results, natural cubic splines of the time with fixed degrees of freedom per year (df = 4) or city-specific degrees of freedom per year were used to control seasonal and long-term trends. Besides that, the exposure was adjusted by natural cubic spline or quadratic B-spline with three internal knots placed at the 10th, 75th, and 90th percentiles of city-specific temperature distributions. In general, the estimated total attributable fractions did not differ statistically between models. However, the uncertainties were greater in the models where the exposure was adjusted by quadratic B-spline.

## 4. Discussion

Our results show a significant association between exposure to temperature and mortality from respiratory diseases in the elderly in the Brazilian capital cities from 2000 to 2017. The associations were observed for both low and high temperatures, depending on the capital’s climatic characteristics. Most capitals had accumulated effects estimated by J or U-shaped exposure–response curves. In general, the associations were stronger, with higher relative risks, for heat. Regarding extreme temperatures in all capitals, except for the South region, extreme heat was more strongly associated with death than extreme cold.

The significant association between exposure to temperature and mortality from respiratory diseases in the elderly has also been found in other studies. In addition to the average daily temperature, other exposure indicators were evaluated, among which are the minimum daily temperature, maximum daily temperature, apparent temperature and diurnal temperature range [25,26,27].

Lin et al. [28] assessed exposure to extremes of temperature and its effects on mortality among the elderly in Taiwanese cities. In contrast to our results, the risks of death from respiratory diseases were higher for low average daily temperatures when compared to high temperatures. In China, a study in Beijing estimated the effects of two-day cumulative exposure (by moving average) to average daily temperatures in the periods of heat (April to September) and cold (October to March) on mortality from respiratory diseases in the elderly aged 65 and over [29]. In the heat period, the risk of death was 8% (95% CI: 1% to 15%) for each 5 °C increase in the two-day temperature average, and in the cold period, the increase was 13% (95% CI: 6% to 20%). Probably, the physiological resilience of individuals should influence these results, as well as past illnesses.

In the elderly, the physiological changes resulting from the aging process may hinder thermoregulation, making the physiological capacity to adapt slower in this group than in younger age groups [10]. Besides that, the elderly have co-morbidities that make them more likely to be susceptible the impacts of extreme temperatures and their variations. Han et al. [8] showed that the elderly are more vulnerable to extreme events such as heat waves than younger groups and that, on the other hand, vulnerability to cold is the same for the entire population regardless of age and sex.

In all regions of Brazil, the fractions of deaths attributable to exposure to temperature suggest an increase in the number of deaths attributable to heat and cold. The total impact of temperature was significant in capitals that have historically experienced extreme temperatures of heat and cold. The temperature range seems to be an important indicator to explain the heterogeneity among cities. In absolute numbers, the impact will be greater in the Southeast region, which concentrates 44% of the Brazilian population, with the capital Rio de Janeiro being the most impacted by the increase in temperature.

In some capitals, mainly in the northeast, the minimum mortality temperature was approximately 26 °C. This explains why the fraction attributable to heat was lower than that of cold. It is worth mentioning that the excess of deaths in the moderate cold is associated with temperatures on average between 22 °C and MMT, except for the cities in the South and Southeast. Although the results point to a higher impact of cold, it is worth mentioning that for Brazil this impact corresponds to exposure to temperatures below 26 °C differently from other places in Europe and China, where minimum temperatures were below 0 °C [6,29]. In some capitals, mainly in the North and Northeast regions, for example, the average daily temperatures were above 20 °C.

Even though the risk of death is greater at extremes of temperature, the health impact is lower in these ranges. The reason is that the frequency of days with extreme temperatures is lower. It is worth noting that with climate change, it is very likely that the temperature distribution will shift to higher temperatures and, therefore, the impact on health will be greater.

The capitals of the North and Northeast regions had a greater impact related to temperature. These are regions with high temperatures all year round and with lower temperature variability than other regions in Brazil, favoring an adaptive response of this population. Thus, as the populations residing in these places may be more used to high temperatures and for longer periods, they would be more adapted to the heat, not evolving to a more fatal outcome. This lower susceptibility to exposure to heat due to the adaptation of populations living in hot regions has already been suggested by some studies [30,31,32].

In any case, this potential adaptive response can also be associated with issues of collective vulnerability, health services and social determinants and conditions [33]. Hacon et al. [34] pointed out a precarious human development condition in the municipalities of the North and Northeast regions and with limited capacity to face climate change. Additionally, these two regions had the worst performance index of the Unified Health System in 2010 (http://i3geo.saude.gov.br/i3geo/sage/abremapa.php?id=1, last access 18 October 2019) and the highest percentage of elderly patients hospitalized in the last 12 months for 24 h or more [35,36]. Therefore, investments will be necessary to expand the response capacity of health services, such as specific outpatient services to assist patients with signs and symptoms resulting from increased temperature. For this, it will be necessary to improve the capacity of health services and the management of health services. Climate change brings new challenges for medical services, impacting mainly on elderly people in marginal living conditions.

Even though Brazil is a complex country that is territorially extensive, multifaceted, with great cultural, socioeconomic, ecological and climatic heterogeneity, the life expectancy of Brazilians is on an increasing trend. It is estimated that between 2010 and 2050, the elderly population will triple in the country, increasing the burden of disease [37]. Synergistically with an increase in life expectancy, temperature variability can intensify pre-existing morbidities and cause complications from flu and allergies, such as pneumonia, asthma, and chronic obstructive pulmonary disease (COPD), particularly on smoking people. In this sense, the results present the human health dimensions and can be strategic for the organization of health services, such as the elaboration of a care protocol, the training of health professionals and actions that favor the mitigation and/or adaptation of the population in the occurrence of extreme events. Additionally, measures and actions that improve the quality of homes are needed, to improve air circulation and, when possible, propose the use of environmental cooling equipment. In other words, this study highlights the need to develop effective policies and to mobilize public engagement to improve health services for the elderly in Brazil, because the strengthening of public health services needs to be a central component of adaptation to climate change.

This study has some limitations, including the lack of adjustment of risk estimates for air pollutants and infectious diseases, such as influenza epidemics that are expected in cold months, mainly in the Southern region. However, these variables apparently act as mediators of the causal pathways between temperature and mortality for respiratory diseases [38]. At any rate, some studies have already studied the role of these variables in the relationship between temperature and health outcomes [14,39,40]. O’Neil et al. [30], for example, showed that the associations between climate and health persisted even with control of air pollution and respiratory epidemics, but risk assessment and adaptation programs to climate change are better when the analyses consider these factors. In addition, the risk of mortality from respiratory diseases was higher at high temperatures, when the level of pollution (PM_10_) was around 60 μg/m^3^, as shown by Pinheiros et al. [14]. Additional, one should consider synergic effects, such as high temperatures raising the levels of ozone and other air pollutants that exacerbate respiratory disease.

## 5. Conclusions

Our results show a significant impact of non-optimal temperature on the respiratory health of the elderlies living in Brazil. The associations were observed for both low and high temperatures, depending on the city’s climatic characteristics. Regarding extreme temperatures, except for the South region, extreme heat was more strongly associated with death than extreme cold. Our results may contribute to national climate change policy, because the health impact of non-optimal temperatures on the elderly is evidenced, which allows the decision makers to implement proactive action related to respiratory diseases in the cities that have critical temperature variations. Considering the diversity of the various regions of the country, experimental studies seeking signs and early symptoms of the impact of temperature on the health of the elderly, both from heat and cold, are recommended.

## Figures and Tables

**Table 1:** Descriptive statistics, total number of deaths and temperature distribution, by state capitals and the entire Brazil, 2000 to 2017.

Cities	Total Deaths (*n*)	Temperature (°C)					
		Average	Minimum	P25	P50	P75	Maximum
**Midwest**							
Brasilia	12,510	22.1	15.3	20.8	21.9	23.15	29.7
Campo Grande	7544	23.9	8.0	22.3	24.5	26.0	32.1
Cuiaba	4011	25.8	12.6	24.8	25.9	27.1	32.3
Goiânia	11,932	23.4	15.9	22.1	23.2	24.4	31.7
**North**							
Belem	15,569	26.6	23.5	25.8	26.6	27.5	29.4
Boa Vista	970	27.2	22.9	25.7	27.0	28.6	32.2
Macapa	1463	26.1	22.6	25.1	26.0	27.2	29.1
Manaus	8628	26.0	21.9	25.2	25.8	26.7	33.1
Palmas	593	26.2	21.5	25.0	26.0	27.2	33.0
Porto Velho	2344	25.9	17.6	25.0	25.7	26.6	30.6
Rio Branco	2568	25.1	15.0	24.4	25.2	26.1	30.7
**Northeast**							
Aracaju	3735	25.4	21.9	24.5	25.6	26.4	28.7
Fortaleza	19,315	26.7	24.0	26.2	26.8	27.2	29.1
João Pessoa	6199	25.7	22.4	24.8	25.9	26.6	28.6
Maceió	7115	25.1	21.6	24.1	25.2	26	28.5
Natal	6176	25.9	22.6	25.1	26.1	26.7	28.4
Recife	16,599	25.7	22.5	24.7	25.8	26.6	28.9
Salvador	19,442	25.4	20.8	24.3	25.5	26.5	29.1
São Luis	5602	26.9	23.3	26.1	27.0	27.7	29.3
Teresina	5686	27.8	22.7	26.1	27.7	29.5	33.5
**South**							
Curitiba	13,175	17.3	3.3	14.9	17.6	20.1	25.6
Florianopolis	2651	20.7	9.3	18.2	20.8	23.5	29.2
Porto Alegre	14,888	19.5	5.4	16.3	20.0	23.2	31.0
**Southeast**							
Belo Horizonte	20,274	20.4	11.4	18.5	20.7	22.2	28.2
Rio de Janeiro	90,887	23.2	15.0	20.8	23.0	25.5	31.8
São Paulo	121,459	19.4	7.7	17.3	19.6	21.8	27.7
Vitória	1307	23.5	17.3	21.8	23.6	25.2	29.8
Brazil	422,642	24.3	3.3	22.9	25.2	26.6	33.5

**Table 2:** Attributable fraction (AF) of mortality (%) and minimum mortality temperature (MMT) by capital cities and the entire Brazil, 2000 to 2017.

Cities	MMT		Total		Cold		Heat	
	%	°C	AF%	(95% IC)	AF%	(95% IC)	AF%	(95% IC)
Midwest								
Brasília	65	22.6	6.02	(1.56, 10.09)	3.4	(−0.22, 6.47)	2.62	(0.18, 4.77)
Campo Grande	81	26.5	6.84	(−5.48, 16.66)	4.64	(−7.29, 14.88)	2.2	(1.07, 3.20)
Cuiabá	87	28.0	10.69	(−5.56, 22.69)	8.35	(−8.05, 21.53)	2.34	(1.57, 3.02)
Goiânia	66	23.9	8.08	(3.47, 12.76)	4.33	(1.05, 7.54)	3.75	(1.84, 5.60)
North								
Belém	1	24.3	10.06	(−18.81, 29.89)	−0.05	(−0.16, 0.04)	10.11	(−15.26, 29.90)
Boa Vista	33	26.1	3.55	(−5.85, 11.54)	0.85	(−2.67, 3.7)	2.7	(−6.78, 9.49)
Macapá	67	26.8	5.15	(−0.42, 10.77)	4.00	(−3.48, 10.5)	1.15	(−3.38, 4.97)
Manaus	18	25	4.55	(−2.50, 10.8)	0.22	(−0.35, 0.81)	4.33	(−2.95, 10.51)
Palmas	80	27.6	4.12	(−3.30, 10.84)	2.46	(−6.66, 10.78)	1.66	(−1.07, 3.98)
Porto Velho	85	27.2	5.11	(−4.64, 13.45)	3.77	(−7.4, 14.27)	1.34	(0.33, 2.31)
Rio Branco	70	25.9	2.73	(−1.68, 6.37)	1.01	(−2.98, 4.77)	1.72	(−1.7, 4.60)
Northeast								
Aracajú	1	22.8	14.55	(−9.66, 33.21)	−0.05	(−0.14, 0.03)	14.6	(−11.5, 32.00)
Fortaleza	38	26.6	2.91	(−2.55, 7.63)	0.87	(−1.34, 2.93)	2.05	(−3.04, 6.2)
Joao Pessoa	96	27.4	22.95	(8.94, 34.16)	22.84	(8.64, 34.79)	0.11	(−0.14, 0.31)
Maceió	95	26.9	11.53	(−6.16, 25.75)	11.44	(−6.32, 25.42)	0.09	(−0.25, 0.37)
Natal	1	23.8	9.3	(−7.37, 22.18)	−0.06	(−0.17, 0.05)	9.36	(−6.25, 21.97)
Recife	52	25.9	4.96	(0.54, 8.81)	2.12	(−1.4, 5.59)	2.84	(−0.93, 6.31)
Salvador	93	27.3	15.25	(5.04, 24.57)	14.92	(4.09, 23.28)	0.33	(0.01, 0.63)
Sao Luís	93	28.2	12.86	(−3.51, 25.64)	12.61	(−4.67, 25.18)	0.25	(−0.14, 0.63)
Teresina	29	26.4	6.85	(−2.11, 14.35)	0.28	(−2.79, 2.91)	6.57	(−1.37, 13.41)
South								
Curitiba	82	20.8	9.8	(−4.12, 21.13)	9.14	(−4.05, 21.25)	0.66	(−0.18, 1.43)
Florianopolis	84	24.5	11.24	(−0.58, 22.22)	9.82	(−2.68, 20.63)	1.41	(0.82, 1.97)
Porto Alegre	86	24.6	16.36	(4.41, 26.63)	14.65	(1.88, 25.19)	1.70	(1.26, 2.08)
Southeast								
Belo Horizonte	58	21.2	3.7	(−1.20, 8.01)	2.33	(−1.43, 6.12)	1.37	(−1.74, 3.99)
Rio de Janeiro	56	23.6	7.91	(4.56, 10.99)	3.33	(0.83, 5.6)	4.57	(2.31, 6.45)
São Paulo	67	21.1	4.99	(1.07, 8.59)	3.64	(−0.84, 7.72)	1.35	(−0.89, 3.55)
Vitória	81	25.6	4.71	(−4.75, 12.65)	4.25	(−5.89, 13.88)	0.46	(−0.68, 1.5)
Brazil	67 *	25.9 *	7.54	(5.36, 9.21)	4.69	(2.94, 6.17)	2.84	(1.45, 3.95)

* Median.

## Data Availability

Publicly available datasets were analyzed in this study. This data canbe found here: https://datasus.saude.gov.br.

## Supplementary Materials

The following are available online at https://www.mdpi.com/article/10.3390/ijerph18115550/s1, Figure S1: Moderate and extreme attributable fraction of mortality (%). Brazil, 2000 to 2017, Figure S2: Total attributable fraction (AF) of mortality (%) according to model adjustment. Brazil, 2000 to 2017, Table S1: Total attributable fraction (AF) of mortality (%), according to model adjustment, by capital cities and the entire Brazil, from 2000 to 2017.
