# Supplementary material for: Mortality Risk from Respiratory Diseases Due to Non-Optimal Temperature among Brazilian Elderlies
Source: Int J Environ Res Public Health. 2021 May 22;18(11):5550. doi: 10.3390/ijerph18115550 (PMC8197018; doi:10.3390/ijerph18115550)
Supplement: Supplementary file 1 [file ijerph-18-05550-s001.zip › ijerph-1138208-supplementary.pdf]

International Journal of
Environmental Research and Public Health

MDPI

Supplementary Materials

Table S1. Total attributable fraction (AF) of mortality (%), according to model adjustment, by capital cities and the entire Brazil, from 2000 to 2017.

Cities	Natural Spline, City-Specific Degrees of Freedom		Natural Spline, Fixed Degrees of Freedom (df = 4)		B-Spline, City-Specific Degrees of Freedom		B-Spline, Fixed Degrees of Freedom (df = 4)	
	**AF%**	**(95% IC)**	**AF%**	**(95% IC)**	**AF%**	**(95% IC)**	**AF%**	**(95% IC)**
Midwest								
Brasília	6.02	(1.56, 10.09)	6.33	(2.91, 9.24)	5.37	(−0.59, 10.56)	5.18	(−0.69, 9.93)
Campo Grande	6.84	(−5.48, 16.66)	5.77	(−3.59, 12.82)	3.75	(−1.71, 7.88)	10.24	(−1.63, 20.59)
Cuiabá	10.69	(−5.56, 22.69)	4.26	(−0.87, 8.79)	8.69	(−6.9, 20.43)	4.41	(−0.16, 7.9)
Goiânia	8.08	(3.47, 12.76)	5.60	(1.21, 10.15)	6.79	(2.82, 10.37)	3.67	(−0.41, 7.24)
North								
Belém	10.06	(−18.81, 29.89)	5.07	(−23.68, 26.09)	11.40	(−12.07, 29.25)	4.94	(−0.87, 9.88)
Boa Vista	3.55	(−5.85, 11.54)	4.08	(−4.49, 11.45)	9.61	(−21.62, 30.13)	5.20	(−4.6, 13.41)
Macapá	5.15	(−0.42, 10.77)	2.31	(−34.57, 29.9)	19.66	(2.35, 31.76)	12.40	(−13.9, 31.9)
Manaus	4.55	(−2.50, 10.8)	2.96	(−1.07, 6.47)	9.16	(1.4, 15.25)	7.39	(0.94, 12.97)
Palmas	4.12	(−3.30, 10.84)	3.47	(−3.23, 8.66)	4.81	(−5.51, 13.37)	5.09	(−4.55, 12.39)
Porto Velho	5.11	(−4.64, 13.45)	5.63	(−0.92, 10.36)	8.63	(−4.36, 18.71)	7.47	(−3.41, 15.25)
Rio Branco	2.73	(−1.68, 6.37)	12.22	(−8.13, 28.09)	3.37	(−5.58, 9.93)	15.07	(−2.98, 28.96)
Northeast								
Aracajú	14.55	(−9.66, 33.21)	9.50	(−26.98, 34.1)	12.81	(−9.18, 29.93)	2.63	(−28.96, 25.66)
Fortaleza	2.91	(−2.55, 7.63)	4.09	(−2.32, 9.66)	4.58	(−1.4, 10.43)	7.09	(−7.29, 19.95)
Joao Pessoa	22.95	(8.94, 34.16)	15.89	(−0.04, 29.25)	23.49	(6.79, 36.07)	12.95	(0.39, 22.12)
Maceió	11.53	(−6.16, 25.75)	11.74	(−6.13, 24.76)	13.68	(1.72, 23.26)	8.89	(−3.78, 20.2)
Natal	9.3	(−7.37, 22.18)	11.01	(−7.97, 25.6)	9.25	(3.12, 14.63)	5.25	(−4.3, 13.55)
Recife	4.96	(0.54, 8.81)	9.03	(−16.83, 28.85)	7.22	(−22.29, 28.09)	18.66	(−0.76, 36.43)
Salvador	15.25	(5.04, 24.57)	14.76	(2.74, 24.23)	13.54	(−0.37, 24.81)	15.65	(2.87, 27.09)
Sao Luís	12.86	(−3.51, 25.64)	13.32	(−3.66, 26.57)	10.10	(−2.44, 20.58)	11.01	(−1.68, 22.91)
Teresina	6.85	(−2.11, 14.35)	6.34	(−44.95, 38.8)	14.47	(−19.96, 36.86)	16.85	(−18.94, 38.79)
South								
Curitiba	9.8	(−4.12, 21.13)	8.87	(−6.79, 21.7)	15.93	(1.68, 27.88)	13.10	(−0.34, 24.73)
Florianopolis	11.24	(−0.58, 22.22)	7.20	(2.94, 10.18)	10.18	(−1.56, 19.37)	4.97	(3.13, 6.17)
Porto Alegre	16.36	(4.41, 26.63)	13.54	(−0.54, 24.81)	13.40	(1.59, 23.83)	10.11	(−4.79, 22.5)
Southeast								
Belo Horizonte	3.7	(−1.20, 8.01)	4.55	(1.31, 7.68)	2.13	(−5.31, 8.88)	3.22	(−0.61, 6.57)
Rio de Janeiro	7.91	(4.56, 10.99)	5.72	(3.72, 7.57)	6.74	(3.87, 9.31)	5.24	(2.7, 7.51)
São Paulo	4.99	(1.07, 8.59)	3.81	(1.12, 6.37)	8.42	(−1.72, 16.85)	4.24	(−3.79, 11.52)
Vitória	4.71	(−4.75, 12.65)	2.72	(−0.87, 5.65)	6.08	(−6.89, 16.81)	3.10	(−4.18, 8.58)
Brazil	7.54	(5.36, 9.21)	6.37	(4.14, 8.05)	8.56	(5.15, 11.23)	6.9	(3.88, 9.35)

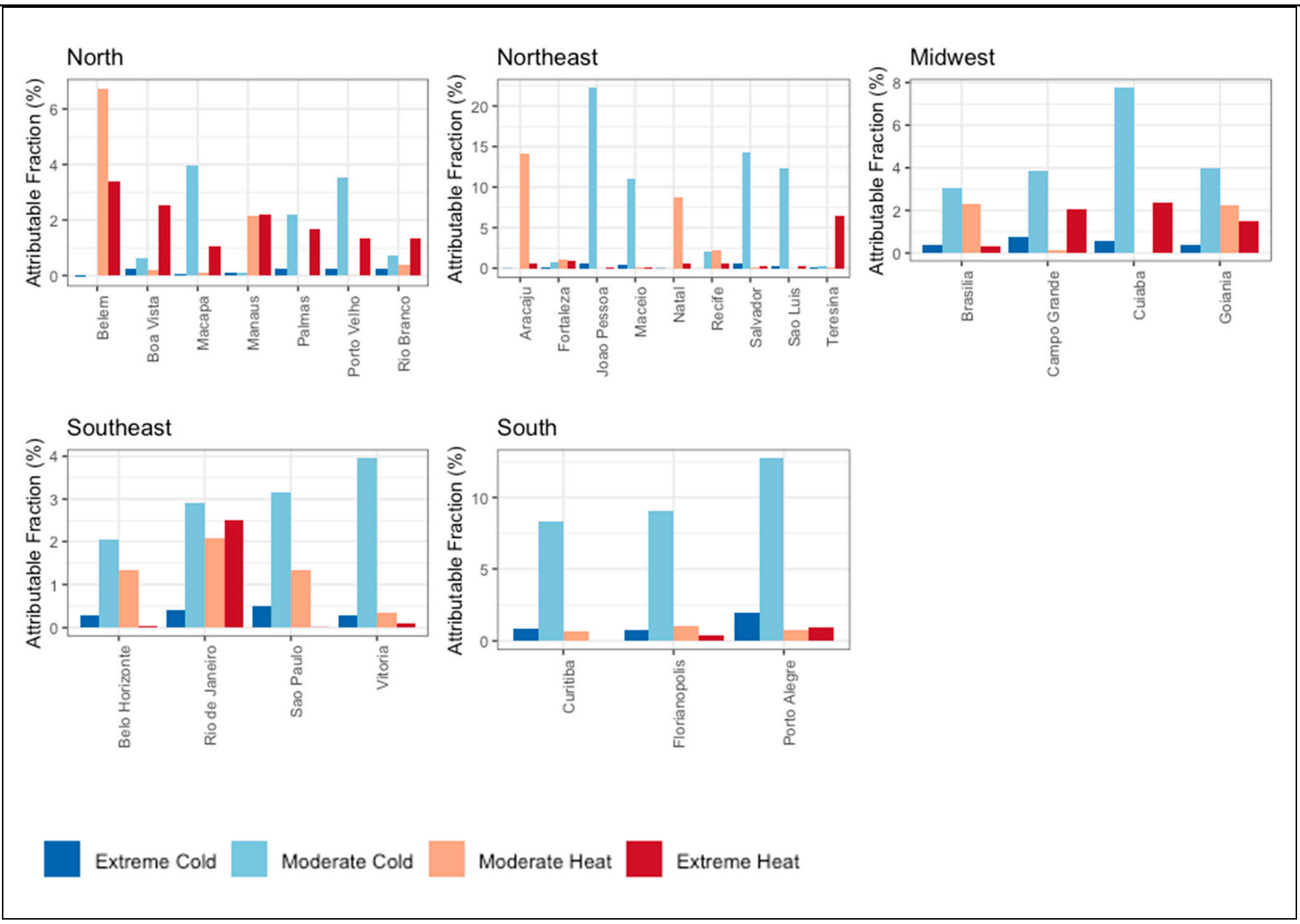

Figure S1. Moderate and extreme attributable fraction of mortality (%). Brazil, 2000 to 2017.

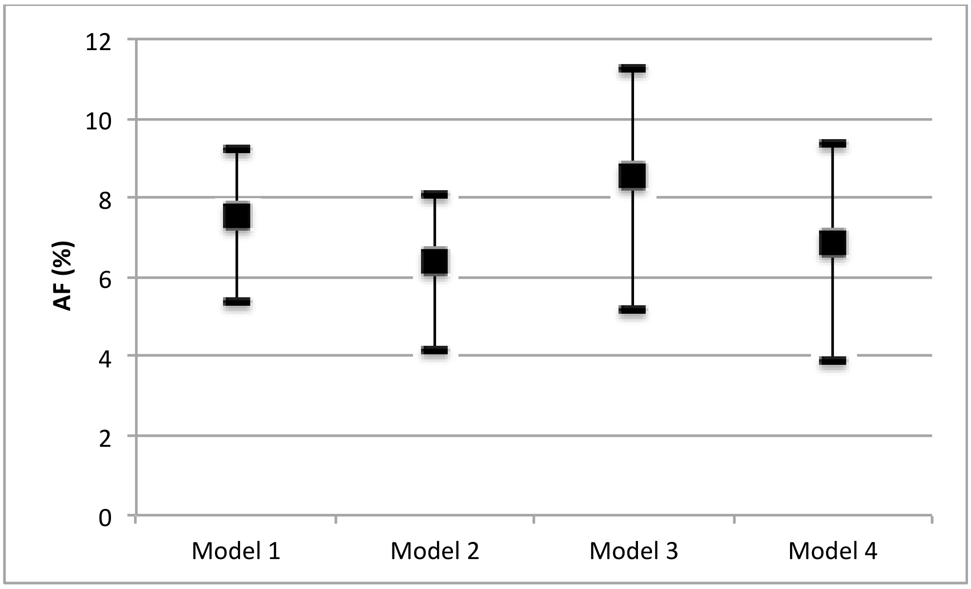

Figure S2. Total attributable fraction (AF) of mortality (%) according to model adjustment. Brazil, 2000 to 2017. Note: Model 1: Natural spline for exposure and city-specific degree of freedom for trend and seasonality; Model 2: Natural spline for exposure and four degrees of freedom for trend and seasonality; Model 3: quadratic B-spline for exposure and city-specific degree of freedom for trend and seasonality; Model 4: quadratic B-spline for exposure and four degree of freedom for trend and seasonality.
